# Gene expression analysis of primordial shoot explants collected from mature white spruce (*Picea glauca*) trees that differ in their responsiveness to somatic embryogenesis induction

**DOI:** 10.1371/journal.pone.0185015

**Published:** 2017-10-02

**Authors:** R. G. Rutledge, D. Stewart, C. Overton, K. Klimaszewska

**Affiliations:** Natural Resources Canada, Canadian Forest Service, Laurentian Forestry Centre, 1055 du P.E.P.S., Stn. Sainte-Foy, Québec, Canada; Defense Threat Reduction Agency, UNITED STATES

## Abstract

Within a plantation of clonal somatic embryo-derived white spruce trees that belonged to four genotypes, one genotype (G6) has consistently responded for the last 16 years, to the induction of somatic embryogenesis within primordial shoot explants. Analysis of fourteen individuals within this genotype subsequently revealed a group of clonal trees that were nonresponsive. This in turn provided a unique opportunity to conduct differential gene expression analysis in the absence of genotype-specific factors. Absolute qPCR was first used to expand the analysis of several genes previously identified via microarray analysis to be differentially expressed during SE induction, along with the inclusion of two nonresponsive genotypes. While this demonstrated a high level of repeatability within, and between, responsive and nonresponsive genotypes, it did not support our previous contention that an adaptive stress response plays a role in SE induction responsiveness, at least with respect to the candidate genes we analyzed. RNAseq analysis was then used to compare responsive and nonresponsive G6 primordial shoots during the somatic embryogenesis induction treatment. Although not analyzed in this study, this included samples of callus and embryonal masses previously generated from G6 explants. In addition to revealing a large number of differentially expressed genes, de novo assembly of unmapped reads was used to generate over 25,000 contigs that potentially represent previously unidentified transcripts. This included a MADS-domain gene that was found to be the most highly differentially expressed gene within responsive shoot explants during the first seven days of the induction treatment.

## Introduction

Discovery of the ability to induce somatic embryogenesis (SE) from conifer zygotic embryos in 1985 [1,2] has subsequently become an important technology for the clonal propagation of genetically improved seed families at a massive scale [3–5]. Moreover, the recent progress in integration of the forward genomic selection in conifer breeding would greatly benefit from the ability to clone individual trees via SE induction within vegetative explants of mature trees [6]. However, the ability to induce SE in vegetative explants has been restricted to young seedlings of white spruce or young Norway spruce trees [4,7]. Indeed, the paucity of responsive explants has proven to be a major limitation for not only advancing SE induction capabilities, but also in providing supporting evidence that it is even possible to induce SE within explants collected from mature conifer trees [8–10].

Establishment in 2003 of a plantation of three years-old somatic embryo-derived white spruce trees representing clones of four genotypes, provided a reliable source of pre-flush shoot buds for yearly SE inductions [11]. This revealed that primordial shoots (PS) of one of these clonal lines (G6) have remained responsive to SE induction up to the age of 16 years [12]. Subsequent microarray analysis comparing this responsive genotype (G6) with a nonresponsive genotype (G12) revealed differential expression of several stress-related genes, suggesting that SE induction responsiveness could be associated with an adaptive stress response [13]. However, it was not clear as to what extent genotype-specific factors played a role.

The present study extends this earlier work by exploiting the availability of responsive and nonresponsive G6 trees to eliminate genotype-specific factors, in addition expanding the breadth of the analysis by inclusion of two nonresponsive genotype (G12 and G2). Absolute qPCR was utilized to examine the expression of six of these stress-related genes during SE induction treatment of this large cohort of vegetative explants. Unexpectedly this failed to support our earlier contention that broad differences in response to tissue culture imposed stress, are associated with SE induction responsiveness [13].

To further investigate genes associated with SE induction responsiveness, a second induction series was conducted in which the transcriptomes of responsive and nonresponsive G6 explants were compared using RNAseq analysis. In addition to utilizing the Arborea EST-based catalog, Cluseq 3.3, for read mapping [14], this included construction of a second transcript library, generated by de novo assembly of over 1.0x10^8^ reads that did not map to the Arborea EST catalog. This generated over 25K contigs that presumably represent transcripts not present in the Arborea EST catalog.

Applying the empirical analysis of differential gene expression tool within the CLC Genomics Workbench, a large number of differentially expressed genes were identified, albeit most with unknown function. However, this did include differential expression within responsive explants, of a conifer-specific dehydrin, DHN1, previously identified using microarray analysis [13], confirming a potential role in promoting SE induction. In addition, a putative MADS-domain gene within the de novo transcript library was found to be the most highly differentially expressed gene within responsive PS during the first seven days of the SE induction treatment, suggesting that it may play an early role in establishing SE induction responsiveness.

## Results

### Characterization of primordial shoots that are responsive to SE induction

Central to the ability to examine the molecular events associated with SE induction was the identification of a clonal line of SE-derived white spruce trees whose PS are responsive to SE induction. Note that a detailed description of this multi-year project has recently been published [12], in which four clonal lines of somatic trees (G1, G2, G6 and G12) were established in a plantation near Valcartier, Quebec, Canada in 2003. SE induction treatments were conducted during each subsequent year, revealing that PS from G6 have remained responsive into 2016 (Klimaszewska, unpublished). An additional revelation subsequently came from screening of individual G6 trees, which demonstrated that seven of the fourteen G6 trees were nonresponsive [12]. This in turn provided the opportunity to conduct an intra-genotype comparison, with the objective of expanding the previous microarray-based analysis that had compared gene expression of G6 PS explants with those collected from a nonresponsive genotype, G12 [13].

### qPCR gene expression analysis based on sample-specific normalization factors

With the primary objective of examining the reproducibility of candidate gene expression identified during an earlier microarray-based study [13], an induction series was initiated in the spring of 2014 that in addition to G12 included a second nonresponsive genotype, G2. Importantly, this also provided the opportunity to compare gene expression profiles from responsive (G6) and nonresponsive (G6NR) G6 explants that would eliminate any genotype-specific factors. This large cohort of samples, consisting of four explant types sampled at five induction time points, were first used to test the efficacy of sample-specific normalization, based on differences in reference gene transcript quantity relative to its average across each respective sample series.

**Fig 1:**
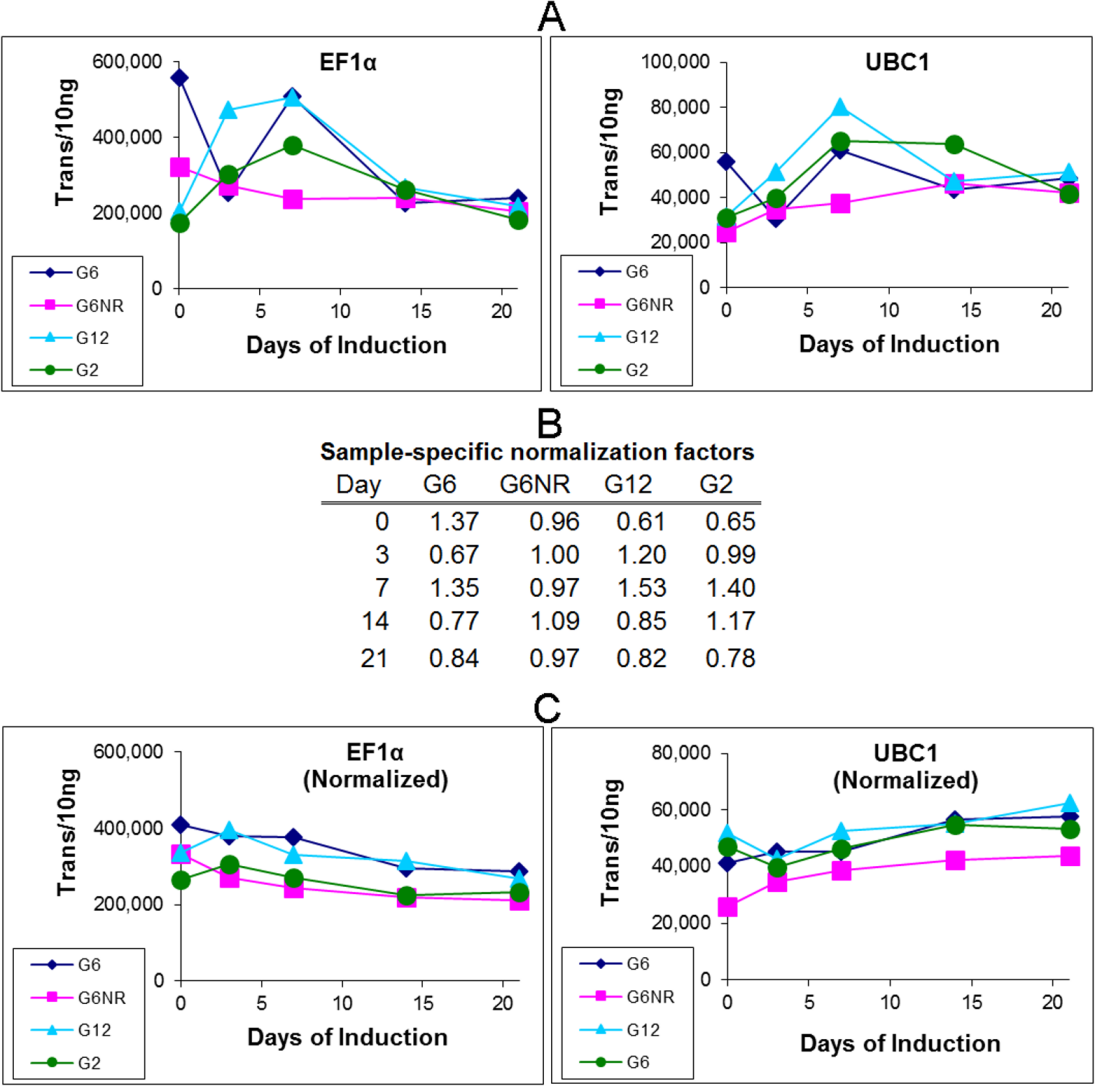
Intra-series normalization based on sample-specific differences in reference gene transcript quantity. (A) Absolute transcripts quantities of two reference genes at five induction time points for each of the four explant types (G12 and G2: nonresponsive genotypes, G6 and G6NR: responsive and nonresponsive G6 trees, respectively). (B) Sample-specific normalization factors were generated by first converting reference gene transcript quantities within each individual sample into a fractional value relative to their respective sample series’ average, followed by averaging the two reference gene values. (C). Reference gene transcript quantities from A were divided by each respective sample-specific normalization factor from B to generate normalized quantities. Note that a central attribute of this approach is that normalization can be applied without loss of absolute scale.

Based on an extensive analysis of nine candidate references genes, it was previously determined that elongation factor (EF1α) and ubiquitin-conjugating enzyme (UBC1) have high levels of expression stability within primordial shoot explants over a 21 day SE-induction treatment [13]. Absolute quantification within each of the four-sample series generally confirmed this, although variances within the first three time points were evident for both reference genes (Fig 1A).

Normalization was initiated by first averaging reference gene transcript quantities across the five samples within each respective explant series. This in turn allowed reference gene transcript quantities within each individual sample to be converted into a fractional value, relative to their respect sample series’ average. Finally, the values for the two reference genes were averaged within each individual sample, to generate a sample-specific normalization factor that reflects the combined divergence of the two reference genes, relative to the sample series average (Fig 1B). The central premise of this approach is that sample-to-sample differences in reference gene transcript quantity is primarily reflective of technical variance associated with sample preparation (i.e. RNA extraction, quantification and reverse transcription).

To provide some perspective, this revealed that divergence in reference gene expression across the twenty samples ranged from 67% to 135%, suggesting a maximal variance of roughly ±34%. Note that this is most certainly an overestimate of the variance associated with sample preparation, in that it includes the technical variance generated by the qPCR quantification, in addition to true differences in reference gene expression. Indeed, this level of variance is similar to the ±25% accuracy previously reported for the LRE-based qPCR methodology used to conduct this analysis [15–17]. Nevertheless, this level of sample-to-sample variance is well within the broadly accepted view that a difference in expression should be >1-fold to be considered biologically significant.

To test the general efficacy of this approach, the raw transcript quantities presented in Fig 1A were normalized by dividing each value by the respective sample-specific normalization factor (Fig 1B). Indeed, this was found to substantively reduce quantitative variance, as best exemplified by the day 0, 3 and 7 samples of G6 and G12 (Fig 1C).

**Fig 2:**
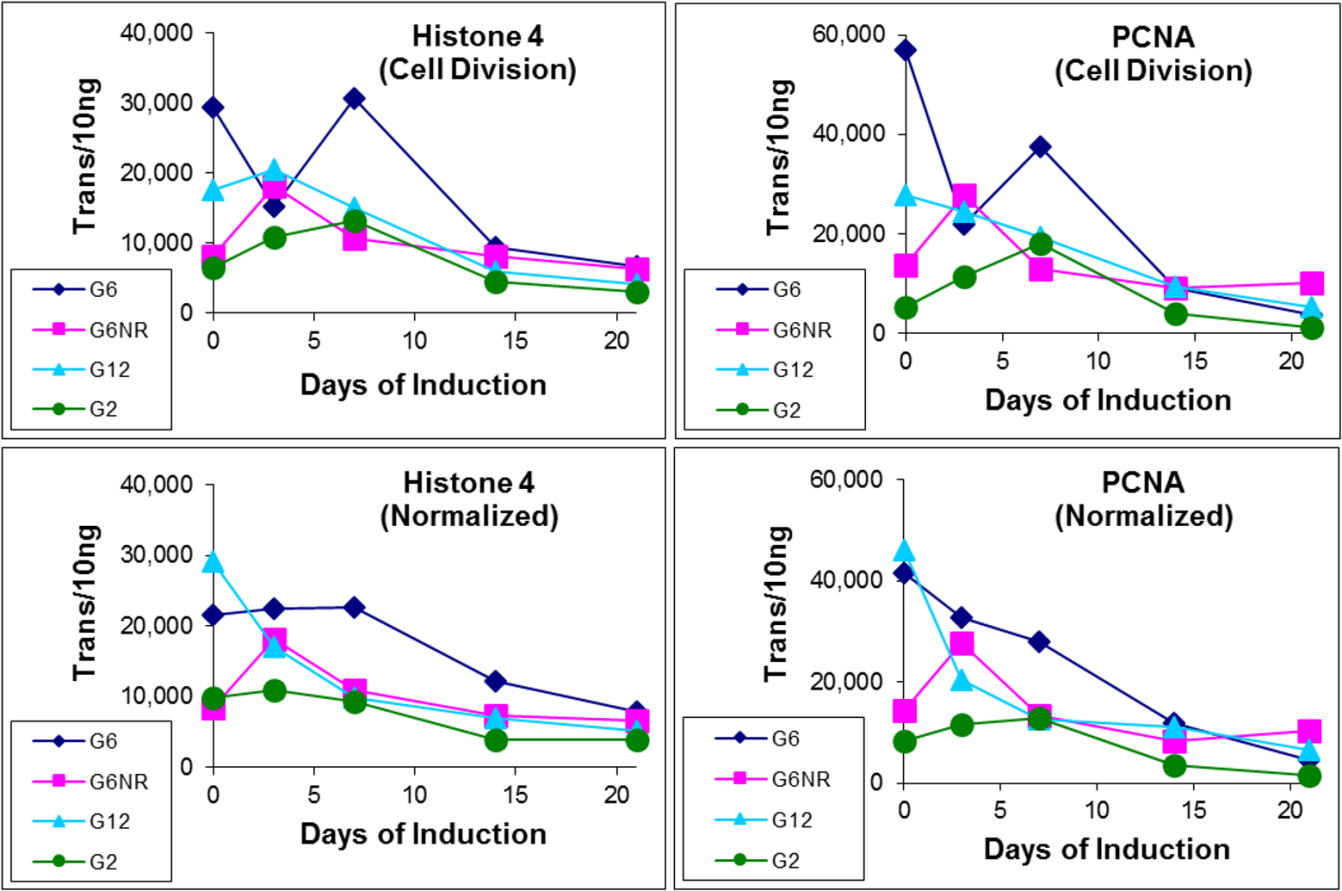
Impact of sample-specific normalization on expression analysis of two cell division genes. As described in Fig 1, sample-specific normalization was found to reduce transcript quantity variance, particularly in the three earliest time points of the G6 and G12 sample series. PCNA: Proliferating Cell Nuclear Antigen.

In order to further assess the efficacy of sample-specific normalization, transcripts from two cell division genes were quantified, which also provided insights into the physiological response to the induction treatment. Consistent with that observed for reference gene expression, this had the greatest impact on transcript quantification within the three earliest times points, revealing a progressive, albeit modest, reduction in cell division throughout the induction period, with some inter-genotype differences being apparent (Fig 2).

### Expression analysis of two stress-inducible genes encoding cytosolic peroxidase homologues

**Fig 3:**
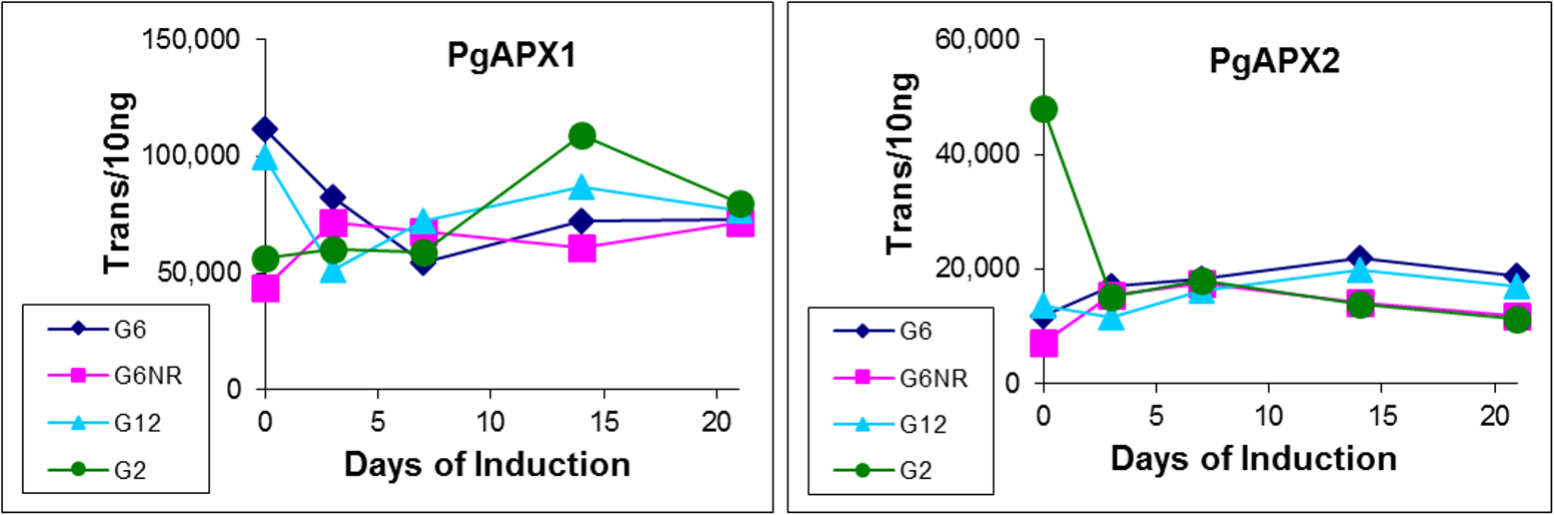
Expression profiles of *P*. *glauca* homologues of APX1 and APX2. Based on the pivotal role in responding to oxidative stress, and taking advantage of a high level of evolutionary conservation within spruce, these two putative members of the ascorbate peroxidase gene family were selected for expression profiling. Note transcript quantities have been normalized as described in Fig 1 using sample-specific normalization factors.

To further explore physiological responses during SE induction, expression of two oxidative stress-inducible genes encoding for ascorbate peroxidase were examined [18,19]. Identified during an extensive search for stress-inducible conifer genes, with the objective of assessing a potential link between stress response and SE induction, spruce was found to possess a small ascorbate peroxidase gene family. Based on extensive amino acid sequence similarity, this subsequently led to identification of putative homologues to the Arabidopsis cytosolic peroxidases, APX1 and APX2. While a detailed description of this gene family is beyond the scope of this study, it was of interest to determine if any explant-specific differences in expression could be identified, in that these two genes have been found to play a central role in abiotic stress response within angiosperms [20,21]. However, no major differences in expression were found either across different sample series or during the induction treatment (Fig 3).

### Analysis of G12- and G6-inducible genes

Availability of these four explant series further provided an opportunity to expand the expression profiling of six differentially expressed genes (Prx52, Prx21, PI20a, PI20b, DHN1, QT repeat) proposed to have a role in determining SE induction responsiveness, identified previously via microarray analysis [13]_._ Note that two other genes (cwInv1 and proline-rich) were not included in this analysis as their differential expression over the 21-day induction period was not as definitive.

**Fig 4:**
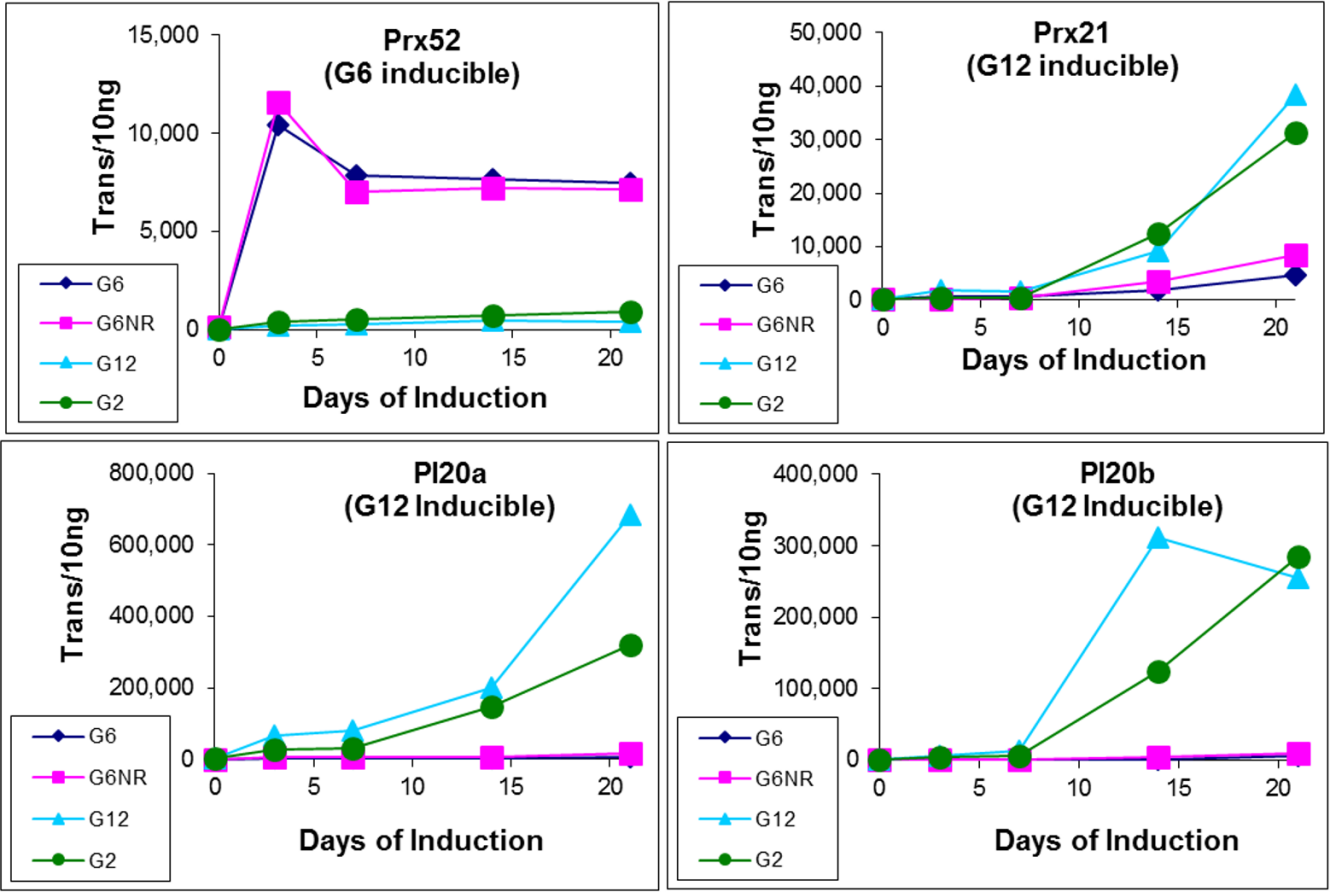
Expression profiling of four candidate genes (Prx52, Prx21, PI20a and PI20b). Previously proposed to be correlated with SE-induction responsiveness, these expression profiles failed to provide support for this supposition. Note that transcript quantities have been normalized using sample-specific normalization factors as described in Fig 1.

As shown in Fig 4, four of these candidate genes provided the most conclusive outcome, which unexpectedly failed to support the previous contention that their expression was correlated to SE induction responsiveness. Indeed, the expression profiles of all four genes were particularly striking in that the level of inter-series correlation was remarkable, not only for the level of repeatability to that observed during the previous SE induction study from which these genes were identified [13], but also for generating near identical profiles for the two G6 explants types (G6 vs. G6NR) and the two nonresponsive genotypes (G2 vs. G12).

Based on genotype-specific induction, it was previously surmised that lack of SE induction within G12 was associated with a hyper-stress response, whereas SE induction within G6 was associated with an adaptive stress response that occurred within the first seven days of induction [13]. Nevertheless, while the expression profiles for G6 and G12 observed here are nearly identical to that observed in this earlier study, the profiles of G6 and G6NR were nearly identical for all four genes, contradicting the supposition that these genes are directly associated with SE induction responsiveness (Fig 4).

**Fig 5:**
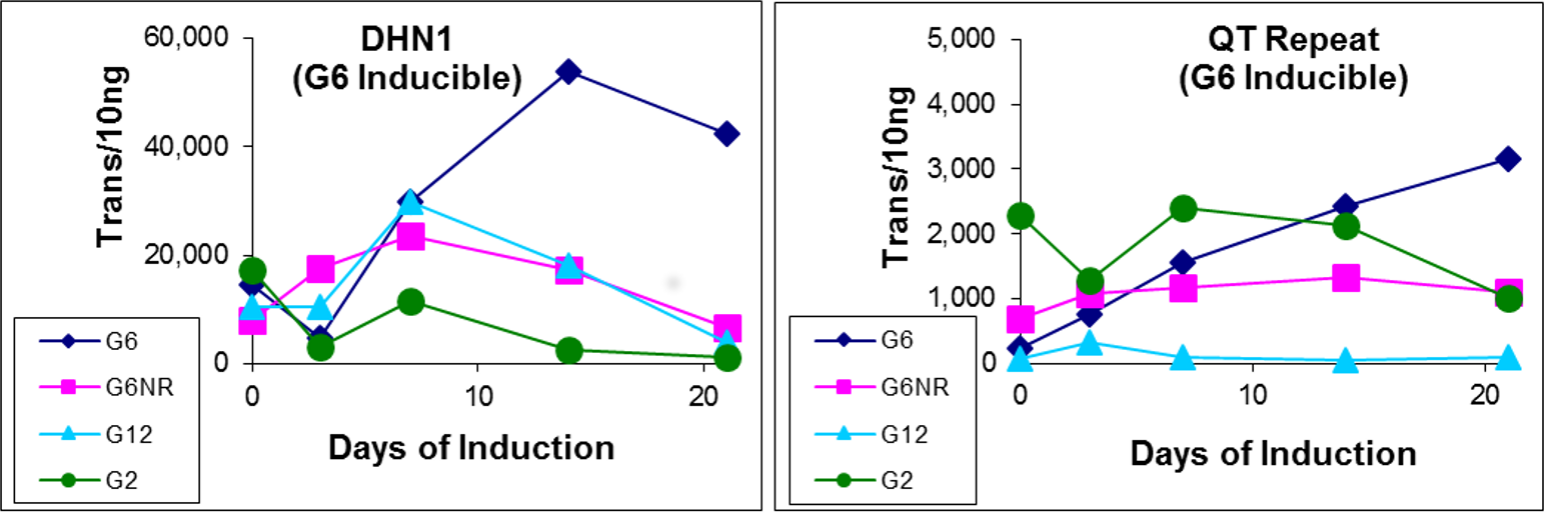
Expression profiles of a conifer-specific dehydrin (DHN1) and a unique QT-repeat gene. This revealed little correlation with SE-induction responsiveness within the first seven days of treatment thought to be a critical period for establishing the induction process. Note transcript quantities have been normalized using sample-specific normalization factors as described in Fig 1.

Although analysis of the two other G6-inducible genes (DHN1 and QT repeat) was less definitive, it also revealed a general lack of differential expression between G6 and G6NR explants during first week of the induction treatment (Fig 5). That is, notwithstanding late stage induction of DHN1 within the responsive G6 explants, overall these profiles failed to provide any substantive support for our earlier supposition that an adaptive stress response plays an important role in SE-induction responsiveness [13]. Instead, the differential expression observed previously is most certainly due to genotype-specific factors that are unlikely related, at least directly, to establishing SE induction responsiveness, with the possible exception of DNH1.

### RNAseq analysis of responsive and nonresponsive G6 explants

To exploit the opportunity to eliminate genotype-specific factors, transcriptome analysis was conducted on samples collected from a second induction series using primordial shoots from responsive and nonresponsive G6 trees. This encompassed three biological replicates collected from four time points (day 3, 7, 15 and 21), totalling 24 samples. Although not analyzed in this study this also included three replicate samples of nonembryogenic callus and embryonal masses generated by G6 explants during an earlier SE induction experiment. RNAseq reads were initially mapped to the Arborea white spruce gene catalogue, Cluseq 3.3, containing 27,270 entries generated from an extensive analysis of EST clones [14]. Referred to as the Cluseq dataset, this successfully mapped 73.3% of all reads, which in turn failed to map about 1.0x10^8^ reads.

Based on the presumption that some of these unmapped reads were generated from transcripts absent from the Cluseq EST catalog, they were subjected to de novo assembly using the CLC Genomics Workbench, with the minimum contig size set to 500 bp. A total of 62.2% of these unmapped reads were successfully assembled into 25,236 contigs (Resource 1, TSA accession GFBZ00000000), which were then used for a second round of read mapping, referred to as the De Novo dataset. To assess the general quality of the de novo assembly, the top ten differentially expressed de novo contigs within the responsive explants from day 3 (described below), were blasted against both Norway spruce (Ver. 1.0, complete) [22] and white spruce (PG29-V4.0) [23] genome assemblies using the genome blast function available on the Congenie.org website. This generated near identical results in which corresponding exonic segments consisting of 94–99% sequence similarity were identified across all ten contigs (S1 File), providing supporting evidence for the effective quality of this de novo assembly.

Identification of differentially expressed genes (DEG) using a tagwise dispersion p-value of <0.05 (S2 File) revealed several notable differences between the Cluseq and De Novo datasets for DEG >2.0-fold differences (S1 File), the most evident being a substantially higher number of DEG identified within the De Novo dataset (Table 1). Additionally, the overall expression level of DEG as based on the average RPKM, while generally similar across the two datasets, were dramatically higher within the responsive buds at day 15, particularly for the De Novo dataset (bolded, Table 1). Although this phenomenon was not characterized further, the timing of this high level DEG expression does closely correlate with the formation of nodule-like structures within G6 explants, which have been associated with EM formation as has been described previously [11,12].

**Table 1:** The number and average expression levels of differentially expressed genes within responsive and nonresponsive G6 primordial shoots.

		Cluseq		De Novo	
	Bud	Total	Av.	Total	Av.
Day	Type	DEG	RPKM*	DEG	RPKM*
3	Resp	24	25.42	81	9.37
	Nonresp	5	9.39	137	4.02
7	Resp	30	29.07	98	33.02
	Nonresp	29	28.16	149	10.34
15	Resp	37	**150.67**	148	**695.88**
	Nonresp	13	5.25	101	2.66
21	Resp	64	41.38	114	13.50
	Nonresp	29	72.49	605	5.08

*RPKM, reads per kilobase per million reads mapped

**Fig 6:**
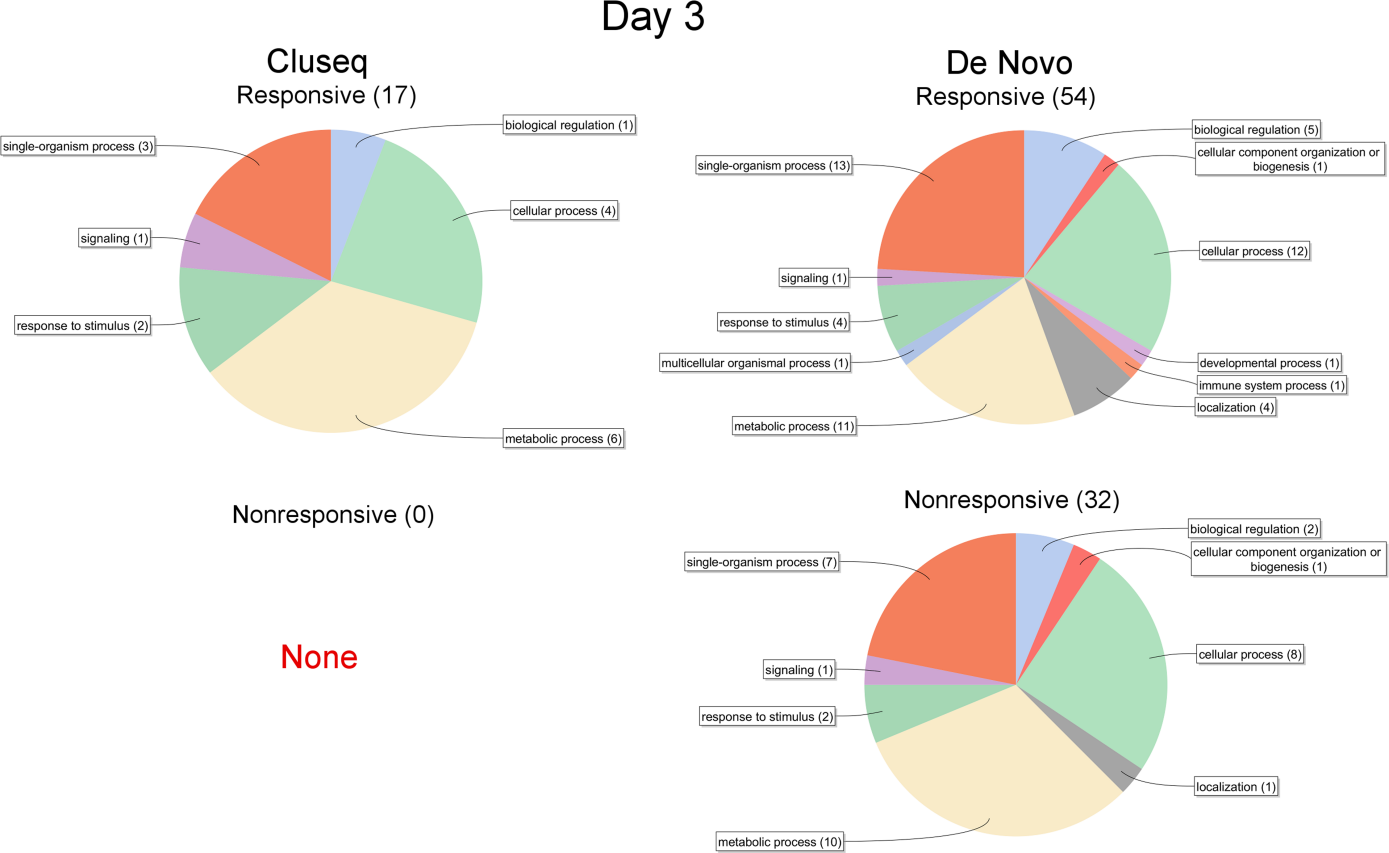
Blast2GO functional annotation of Cluseq and De Novo DEG expressed >2-fold at day 3. The total number of transcripts that were successfully annotated are bracketed.

**Fig 7:**
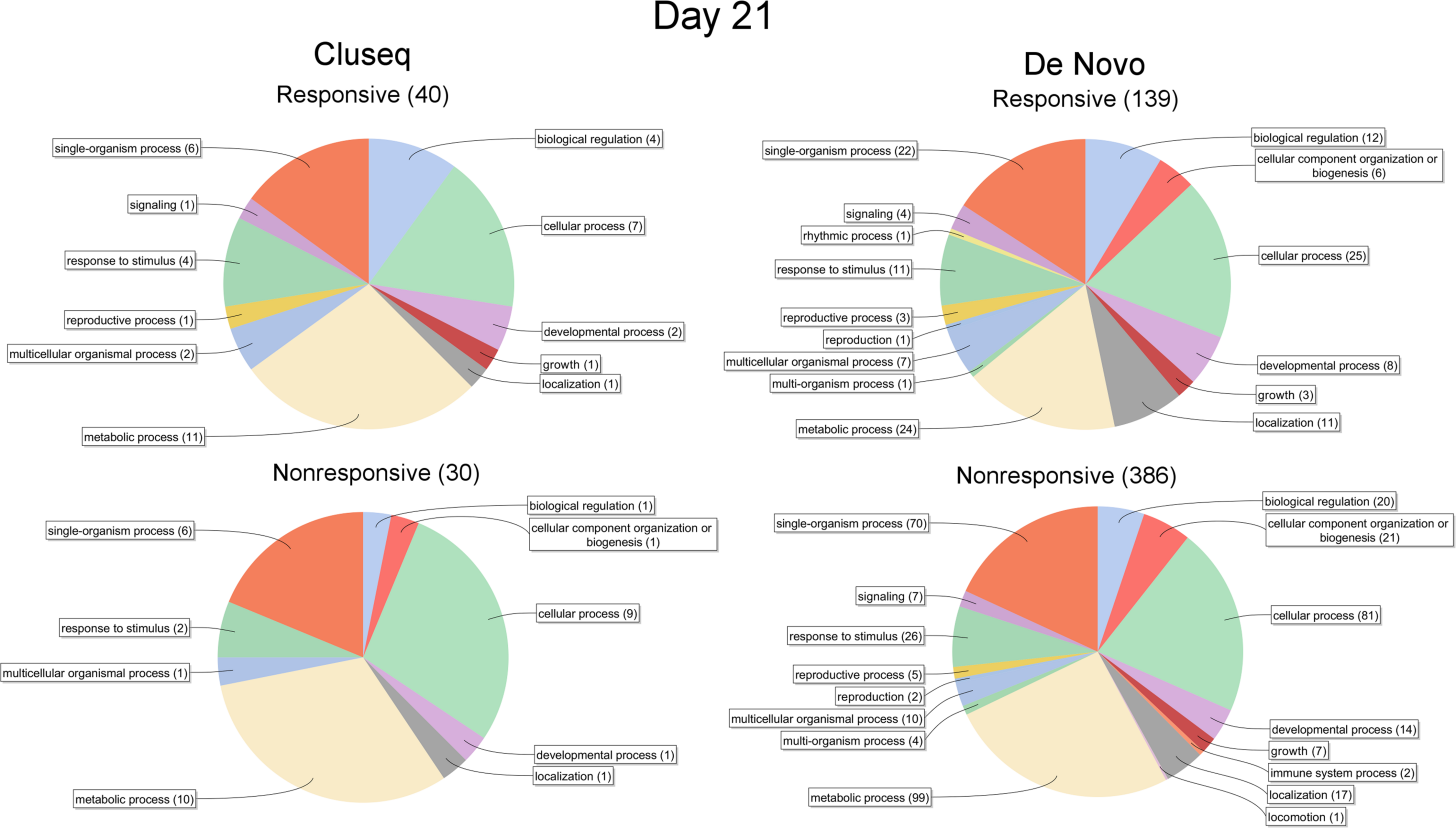
Blast2GO functional annotation of Cluseq and De Novo DEG expressed >2-fold at day 21. The total number of transcripts that were successfully annotated is bracketed.

To assess the functional composition of DEG expressed >2.0-fold at day 3 and 21, functional annotation was conducted using BLAST2GO [24,25]. This was based on the presumption that differences between responsive and nonresponsive buds would be most evident at the two most extreme time points. However, although small differences were apparent between the Cluseq and De Novo datasets, and between day 3 vs. day 21, no prominent differences were evident between responsive and nonresponsive explants at either time point (Figs 6 and 7).

### Functional identification of two of the most highly differentially expressed genes within responsive explants

A detailed examination of the proteins encoded by the ten most highly differentially expressed genes within responsive explants, conducted for both datasets, revealed that the two most differentially expressed genes encode for peptides with known functions. The first was within the Cluseq dataset, which was found to encode for the candidate gene DHN1, which as described earlier, was initially identified during microarray analysis comparing explants from G6 and G12 [13]. Based on the average RPKM values (S2 File), DHN1 was differentially expressed within responsive explants over the entire induction treatment with the highest levels of differential expression at day 21 (Table 2). Indeed, this is similar to that observed for the qPCR analysis conducted on the first induction series (Fig 5), providing further evidence that DHN1 may play a role in SE induction responsiveness.

**Table 2:** RNAseq expression analysis of DHN1 within responsive and nonresponsive G6 buds.

Day	Resp RPKM*	Nonresp RPKM*	FoldDiff.	Tagwise Dis.p-value
**3**	19.94	4.76	4.2	6.13x10^-8^
**7**	17.88	3.49	4.9	6.42x10^-6^
**15**	19.36	2.87	6.8	7.03x10^-10^
**21**	40.39	2.02	18.2	8.94x10^-20^

*RPKM, reads per kilobase per million reads mapped

The second was contig 9846 within the De Novo dataset, which turned out to be the most highly differentially expressed gene within responsive explants at both day 3 and 7, although its expression became undetectable by day 15, as was confirmed by qPCR analysis (Table 3).

**Table 3:** Expression of de novo contig 9846.

	RPKM*		# Trans/10ng**	
Day	Resp	Nonresp	Resp	Nonresp
3	10.00	0.21	355	4
7	7.86	0.00	193	1
15	0.00	0.00	0	0

*RPKM, reads per kilobase per million reads mapped

**Number of transcripts per 10 ng of RNA as determined by absolute qPCR

**Fig 8:**
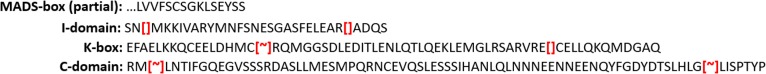
MADS-domain peptide encoded by contig 9846 along with the position of six putative introns. The peptide is divided into the four domains, with the short I-domain being indicative of a type IIc MADS domain protein. Intron position, as determined by blasting Norway and white spruce genome assemblies (S3 File), are designated by bolded red brackets, although the positions of three could not be precisely determined due to alignment anomalies ([**~**]). Accession KY229688.

As summarized in S3 File, blasting contig 9846 against Norway (*Picea abies*) and white spruce genome assemblies generated nearly identical results, producing alignments consisting of seven exons within four genomic contigs, all close to 100% sequence similarity. Moreover, these alignments predicted that the 9846 contig assembly contained three aberrant single base gaps within the putative coding region (all within repetitive base motifs known to be susceptible to reading errors), which when corrected, was found to encode for a 183 amino acid peptide (Accession KY229688). Subsequent GenBank blast analysis revealed that this peptide contains conserved regions indicating that it is a type IIc MADS-domain protein, as based on the short I-domain [26]. Note also that contig 9846 lacks 132 bp within the 5’ of the coding region that encodes most of the MADS domain (Fig 8).

Unfortunately, blast analysis of this putative MADS gene failed reveal any substantive sequence similarity to any conifer EST DNA sequences or their encoded proteins. Blast analysis also failed to reveal any Arabidopsis MADS proteins that share sufficient homology to allow identification of a putative angiosperm homologue.

## Discussion

### Gene expression within vegetative explants during SE induction

With the expectation that SE-derived trees could have a high propensity for SE induction within vegetative explants, a multi-year project was initiated in which SE-derived seedlings generated from four genotypes of embryonal masses were used to establish a plantation. SE inductions were conducted each subsequent year, revealing that primordial shoots from one genotype, G6, have remained responsive [12].

In addition to revealing insights into the morphological aspect of EM formation (discussed further below), this provided the opportunity to conduct transcriptome analysis using microarray analysis, in which G6 explants were compared with that collected from a nonresponsive genotype, G12. This led to the identification of four differentially expressed genes (DEG) within each genotype (referred to as candidate genes) most of which were found to encode homologs of stress-related proteins. This in turn suggested that stress response could be a key aspect underpinning SE induction responsiveness [13].

Identification of nonresponsive PS within individual G6 trees [12] further provided the opportunity to test whether lack of responsiveness was associated with induction of one or more of the four G12 candidate genes, which are supposedly associated with a lack of SE induction responsiveness. Of equal interest was to assess the level of repeatability in candidate gene expression to that observed previously, in addition to that produced in the absence of genotype-specific factors. Inclusion of explants from G12, along with a second nonresponsive genotype, G2, further expanded the analysis. Importantly, this large cohort of samples provided an opportunity to assess the level of technical and biological variance associated with expression analysis based on absolute qPCR, which included testing a reference gene-based normalization methodology that preserves absolute scale.

### An alternative to relative quantification for reference gene normalization

In addition to the many challenges associated with qPCR-based gene expression analysis, the prevalent practice of expressing transcript quantities as fold differences relative to one or more reference genes (aka, relative quantification) limits both the scope and general utility of qPCR [27–29]. Nevertheless, the ability to reduce technical variances associated with RNA extraction, quantification, and reverse transcription arguably makes reference gene-based normalization an essential component for accurate transcript quantification [30]. However, it is equally evident that when applied under a relative quantification paradigm, the efficacy of qPCR becomes seriously compromised. For example, relative quantification does not allow transcript quantities generated by different laboratories, or even across multiple runs within a single laboratory, to be directly compared without the application of some form of universal external standard [31,32]. Among many other limitations, relative quantities are also inherently difficult to interpret, restrict the ability to directly compare transcript quantities across multiple genes and/or samples, and are recalcitrant to the application of basic statistical analysis.

An alternative is to express qPCR-derived quantities as the number of target molecules within a sample, an approach referred to as absolute quantification. Key to the utility of absolute quantification is the universal context that it provides, in that absolute values transcend issues of assay design, instrumentation, and even the type of qPCR data analysis applied, such that it generates quantities that are universally comparable. Although absolute quantification has historically required construction of target-specific standard curves, which in itself introduces significant limitations [33], an alternative approach called LRE qPCR, generates absolute quantities that are target-independent. Based on **L**inear **R**egression of cycle-to-cycle loss in amplification **E**fficiency (LRE), in combination with a universal calibration methodology, LRE qPCR has proven to be a robust method for conducting absolute quantification with high levels of accuracy [13,15–17]. Importantly, a platform-independent software program that automates LRE data analysis further provides the ability to conduct absolute qPCR at an unprecedented scale [17].

In addition to generating quantitative data that is intuitive, absolute quantification also allows reference gene-based normalization to be conducted without loss of absolute scale. A key aspect of this approach is the ability to apply basic mathematics to absolute quantities, such as averaging transcript quantities across multiple samples, which in turn provides the ability to compare transcript quantities within individual samples, to a series-derived average. This in turn generates sample-specific normalization factors, which for this study, were based on sampling four explant types at four time points during SE induction treatment. As described in Fig 1, normalization factors can be derived from multiple reference genes by simply averaging their respective normalization factors generated within each individual sample. The general efficacy of this approach was demonstrated in Fig 1C, in which sample-specific normalization factors were applied to the raw quantities of the two reference genes used to generate them. Application to transcript quantification of two cell replication genes generated similar results, which also revealed a modest but progressive reduction in cell division within all four explant types during the 21-day induction treatment (Fig 2).

### Differences in stress gene expression is not associated with induction responsiveness within G6 explants

To further explore potential differences in stress response, expression of two putative cytosolic peroxidase homologues known to play a central role in oxidative stress response in angiosperms (APX1/2) [18,19] were analyzed (Fig 3). However, this failed to reveal any substantive differences within any of sample types, or across the 21-day induction treatment. Indeed, a lack of correlation between stress response and SE induction responsiveness was further revealed during expression analysis of six candidate genes previously identified via microarray analysis, which generated two broad observations.

The first were high levels of repeatability generated within the expression profiles of all six candidate genes. This included the expression profiles within a second nonresponsive genotype, G2, which were nearly identical to that of G12. Second was that the expression profiles within responsive and nonresponsive G6 explants were also nearly identical (Figs 4 and 5), indicating that the differential expression between the G6 and G12 explants previously revealed by microarray analysis, were most certainly due to genotype-specific factors that are not necessarily related to SE induction responsiveness, as was previously surmised [13].

One potential exception was the conifer-specific dehydrin, DHN1, which was found to be differentially expressed within the responsive G6 explants, characterized by a progressive increase in expression level, particularly during the last two weeks of the induction treatment (Fig 5). Although the significance of this is unclear, a previous study reported induction of DHN1 during establishment of fall dormancy within primordial shoots of Norway spruce, suggesting a role in adapting to drought stress associated with overwintering [34]. However, it remains unclear whether this high expression level of DHN1 truly reflects some form of an adaptive stress mechanism associated with SE induction responsiveness.

### Differential gene expression based on RNAseq analysis

The identification of individual trees within the G6 clonal line that lack SE-induction responsiveness, provided an opportunity to conduct transcriptome analysis in the absence of genotype-specific factors. A second G6 induction series was therefore conducted in which samples collected from four time points were subjected to RNAseq analysis. The resulting reads were first mapped to the EST-based Arborea white spruce gene catalogue, Cluseq 3.3 [14], which successfully mapped 73.3% of all reads.

To determine if any of the resulting unmapped reads were generated from transcripts not present within the Cluseq catalogue, these unmapped reads were subjected to de novo assembly. Surprisingly, this generated over 25K contigs, similar in magnitude to the 27K genes contained within the Cluseq catalog, which initially brought into doubt the quality of the de novo assembly. Nevertheless, this did support the contention that explants cultured *in vitro* under high levels of plant growth regulators used for SE induction, generate expression patterns distinctly different from that within vegetative tissues *in vivo*, from which most of the ESTs within the Cluseq catalogue were derived. Indeed, this De Novo contig catalog subsequently led to the identification of a large cohort of differentially expressed genes (DEG) within the G6 explants that outnumbered that generated by the Cluseq catalog by about ten times (Table 1), which could be indicative of novel nature of the transcripts expressed within explants under SE induction treatment. Unfortunately, functional analysis failed to generate clear functional homologues for the vast majority of the de novo DEG.

A notable exception was a contig within the De Novo catalog that turned out to be the most highly differentially expressed gene within responsive explants at day 3 and 7, which was found to encode for a MADS-domain protein (Fig 8). This is consistent with the presumption that changes in master regulator gene expression during early stages of the induction treatment could be central to establishing induction responsiveness. However, blasting against the Arabidopsis genome failed to identify a potential angiosperm homologue, most certainly exacerbated by the absence of most of the MADS domain due to truncation of the 5’ region within this RNAseq-derived contig.

The general efficacy of the RNAseq analysis was further illustrated by the fact that the most highly differentially expressed gene within the Cluseq dataset, who’s expression progressively increased into the late stages of the induction treatment (Table 2), was found to be DHN1, a conifer-specific dehydrin that was previously identified via microarray analysis [13]. Furthermore, this is consistent with that observed for DHN1 expression within the first induction series based on qPCR analysis (Fig 5), again illustrating the high levels repeatability of differential gene expression within this system.

The general efficacy of the De Novo catalog was further illustrated by the extraordinary high expression levels for DEG at day 15 as based on RPKM values (Table 1). A high level of differential gene expression at day 15 was also observed for the Cluseq dataset, although the 148 DEG identified within the de novo dataset at day 15 generated an average expression level that was nearly 5X higher than that generated by the 37 Cluseq DEG. Although the biological significance of this large burst of differential gene expression is not clear, the timing does correlate with the formation of nodule-like tissues within the responsive explants, which notably, were absent within the nonresponsive explants. This is consistent with that described previously, in that these nodules appear to be a transitionary tissue from which embryonal masses (EM) are generated [11].

Although detailed analysis of other DEG was beyond the scope of this study, these results serve to illustrate the utility of these large RNAseq datasets, which also includes samples of G6-derived EM and callus that were not characterized in this study. Point in case comes from recognition that although advances in understanding the molecular aspects underpinning SE induction have been achieved in angiosperms (reviewed in [35]), effective application to conifers is impeded by their extensive evolutionary divergence. This was illustrated in part by failure to identify an angiosperm homologue to the MADS-domain protein found to be the most highly differentially expressed gene in the responsive explants (Fig 8). Differential expression of the DHN1, a conifer-specific dehydrin, further supports the possibility that fundamental differences exist in the molecular aspects of conifer SE-induction, as compared with angiosperms. Indeed, such a possibility may not be surprising to many in the conifer research community. If this is in fact the case, it would provide support to the fundamental importance of conifer-based research for advancing SE induction technologies, which in turn could be a major contributor to the long-term productivity of the forestry industry.

## Methods

### qPCR expression analysis

RNA extraction, reverse transcription and qPCR amplification were conducted as previously described [13], which also provides the primer sequences used for qPCR expression analysis of the microarray-derived candidate and reference genes. Primer sequences of the five other candidate genes examined in this study are listed in Table 4. Automated LRE analysis was conducted using the latest version of the LRE Analyzer (ver. 9.10) available on the LRE website (sites.google.com/site/lreqpcr/home). Note that detailed descriptions of how LRE qPCR was developed, its performance capabilities, and description of the LRE Analyzer have also been published previously [15–17,36].

**Table 4:** qPCR primer sequences.

Acronym	Unigene	5’ Primer	3’ Primer	Amplicon Size
Histone 4	Pgl.5805	CGTTATCCGTGATGCTGTGACCTACAC	CCTCTTGAGGGCATAGACGACATCC	85 bp
PCNA	Pgl.5975	ACCAAGGCAACTCCGCTGTCT	CATGAACTCGTTCACCAAGGCAACTCC	148 bp
PgAPX1	Pgl.989	GATAAGGCACTGCTTACTGATCCCAGT	ACAGCTATTTCGAGCCAGCTATGAATCTATG	158 bp
PgAPX2	Pgl.383	GCCTTTCTGTCGTTGGTTAGAGTCTGG	AGAGACGGGAGAGCAGTAAGCGTTAG	81 bp
Contig 9846	none	TGAAGTGCAATCTTTGGAAAGTTCAAGTATCC	GCATGATCATGGATAAGTGGGACTAATTAACCC	148 bp

### Sample preparation and RNAseq analysis

Primordial shoot explants samples encompassed three biological replicates collected from four time points (day 3, 7, 15 and 21) resulting in a total of 24 samples. This also included differential expression analysis of three replicate samples of nonembryogenic callus and embryonal masses generated by G6 explants during an earlier SE induction experiment, although an in-depth analysis of these datasets was not conducted in this study. The callus samples were obtained from buds collected from G6 responding trees cultured for 11 weeks on MLV-S medium [11], whereas G6 EMs samples were derived from samples removal from cryopreservation six months earlier and cultured on MLV-S, and subcultured 7 days prior to sample collection. Primordial shoot (PS) samples were collected from responsive and nonresponsive trees cultured for 3, 7, 15 and 21 days on MLV-S. Approximately 80 mg fresh mass of tissue was collected for each sample of callus and EM while approximately 50 mg was collected for each PS sample. All samples were flash frozen in liquid nitrogen and then stored at -80^°^C until RNA extractions were performed.

Tissues was disrupted twice using the Qiagen TissueLyser II at speed 26 for 45 s and total RNA was extracted using the Qiagen Plant Mini Kit (cat. no. 74904) with an on-column DNase digestion using the Qiagen RNase-Free DNase Set (cat. no. 79254) as per the manufacturer’s instructions. RNA extract purity was determined using the Agilent 2100 Bioanalyzer. Detection of contaminating genomic DNA (gDNA) was based on qPCR amplification of EF1a [13] using a 1 μl sample of RNA (100–500 ng depending on the RNA concentration). Samples containing detectable levels of gDNA were DNase treated a second time, followed by a second RNA cleanup procedure again using the Plant Mini Kit, and subjected to another qPCR EF1α analysis to confirm absence of gDNA (<5 genomes per 10 ng RNA).

RNAseq libraries were prepared with the Kapa Stranded mRNA-Seq Kit (Kapa Biosystems) using Ion Xpress barcode adapters (ThermoFisher). The libraries were pooled and sequenced on IonProton sequencers at the Plateforme d’Analyses Génomiques (IBIS, Université Laval, Quebec City, Canada). This generated an average of 1.2x10^7^ reads per sample, producing a total of 3.6x10^8^ reads with an average size between 150 and 200 bases, which have been deposited in GenBank, BioProject accession PRJNA353540.

### Differential gene expression analysis

Following adapter removal and elimination of reads <20 bases, low quality terminal bases were trimmed, resulting in removal of the first 15 bases in addition to trimming reads to a maximum length of 205 bases for samples 1–12 and 170 bases for 13–30. CLC Genomics Workbench (Ver. 8.0.1) was then used to map reads to the Arborea white spruce gene catalogue, Cluseq 3.3, containing 27,270 entries [14], which successfully mapped 73.3% of all reads. Unmapped reads were then subjected to de novo assembly using CLC Genomics Workbench, with the minimum contig size set to 500 bases. A total of 62.2% of these unmapped reads were successfully assembled into 25,236 contigs (S4 File), which was then used for a second round of read mapping.

Expression levels reported as RPKM were subjected to empirical analysis of differential gene expression (EDGE) function within the CLC Genomics Workbench, with tagwise dispersion set to default values and with FDR correction. Results were filtered using a tagwise dispersion p-value <0.05 and sorted using a tagwise fold change ≥2.0-fold for G6 or ≤2.0-fold for G6NR (i.e. ≥2.0-fold induction within each respective phenotype). Blasting ten most highly induced genes for each time point against both the Norway (Ver. 1.0, complete) [22] and white spruce (PG29-v4.0) [23] genome assemblies using the genome blast function available on the Congenie.org website, generated near identical results. That is, corresponding exonic segments consisting of 94–99% sequence similarity, were identified across all contigs (S1 File) for both the Norway and white spruce genome assemblies. Functional annotation of DEG identified at day 3 and 21 was conducted with BLAST2GO using default settings [24,25].

## Supporting information

S1 File Summary of Cluseq and De Novo transcript contigs that were differentially expressed >2.0-fold at each of the four time points. (XLSX)

S2 File Differential gene expression analysis. Generated by the EDGE function within the CLC Genomics Workbench based on RNAseq read mapping to the Cluseq and De Novo contig catalogues for each of the four collection times, in addition to comparison of embryonal masses to callus derived from G6 explants.
(XLSX)

S3 File Blast of contig 9846 against white and Norway spruce genome assemblies. (PDF)

S4 File FASTA sequence file of 25,236 contigs generated by de novo assembly of reads that failed to map to the Cluseq EST library. (FASTA)
