# Supplementary material for: Gene expression analysis of primordial shoot explants collected from mature white spruce (Picea glauca) trees that differ in their responsiveness to somatic embryogenesis induction
Source: PLoS One. 2017 Oct 2;12(10):e0185015. doi: 10.1371/journal.pone.0185015 (PMC5624583; doi:10.1371/journal.pone.0185015)
Supplement: S3 File — (PDF) [file pone.0185015.s003.pdf]

Tree Genetics and Genomes:

Gene expression analysis of primordial shoot explants collected from mature white spruce trees (P. glauca) that differ in their responsiveness to somatic embryogenesis induction"

R.G. Rutledge[1], D. Stewart[1], C. Overton[1], K. Klimaszewska[1]*
1. Natural Resources Canada, Canadian Forest Service, Laurentian Forestry Centre,
1055 du P.E.P.S., Québec, QC G1V 4C7, Canada
*Corresponding author: Krystyna.Klimaszewska@canada.ca, (418) 648-4638

Contig 9846 de novo assembly summary

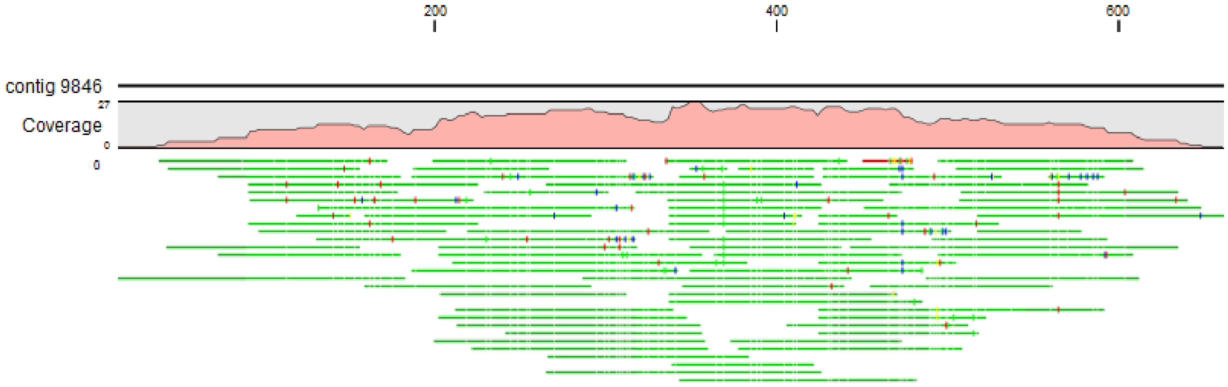

Norway spruce genome assembly blast results

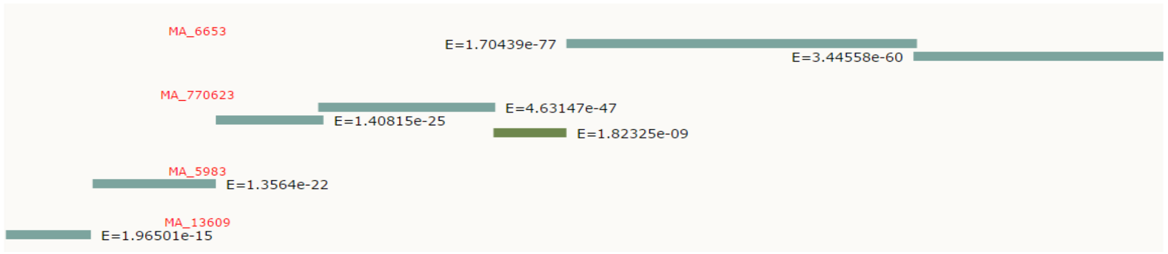

>lcl|MA_13609 len=65589

Length = 65,589

Score = 91.6812 bits (46), Expect = 1.96501e-15
Identities = 49/50 (98%), Frame = +1 / +1

```
Query: 1     CTTGTTGTATTTTCTTGCTCGGGAAAGCTCTCTGAATATTCCAGTTCCAA 50
             ||||||||||||||| ||||||||||||||||||||||||||||||||||
Sbjct: 27776 CTTGTTGTATTTTCTGGCTCGGGAAAGCTCTCTGAATATTCCAGTTCCAA 27825
```

Two single base gaps within repeatative motifs

>lcl|MA_5983 len=34196

Length = 34,196

Score = 115.469 bits (58), Expect = 1.3564e-22
Identities = 72/74 (97%), Gaps = 2/74 (2%), Frame = +1 / -1

```
Query: 51    CACGAGCTTCCAATTCAAA-GATGCTCCACTTTCATTTGAAAAATTC-TATATCTCGCCA 108
             ||||||||||||||||||| ||||||||||||||||||||||||||| ||||||||||||
Sbjct: 25662 CACGAGCTTCCAATTCAAAAGATGCTCCACTTTCATTTGAAAAATTCATATATCTCGCCA 25603


Query: 109   CGATCTTCTTCATG 122
             ||||||||||||||
Sbjct: 25602 CGATCTTCTTCATG 25589
```

>lcl|MA_770623 len=7250

Score = 125.381 bits (63), Expect = 1.40815e-25
Identities = 63/63 (100%), Frame = +1 / -1

```
Query: 122 CCTGCACATATGGTCGAGTTCTTCACATTGCTTTTTCAGCTCTGCGAATTCGCTCTGATC 181
           ||||||||||||||||||||||||||||||||||||||||||||||||||||||||||||
Sbjct: 735 CCTGCACATATGGTCGAGTTCTTCACATTGCTTTTTCAGCTCTGCGAATTCGCTCTGATC 676


Query: 182 CGC 184
           |||
Sbjct: 675 CGC 673
```

Score = 196.746 bits (99), Expect = 4.63147e-47
Identities = 102/103 (99%), Frame = +1 / -1

```
Query: 181 CTCTCTTACGCGAGCAGATCTTAATCCCATTTCAAGTTTCTCCTGCAACGTCTGTAAATT 240
           ||||||||||||||||||||||||||||||||||||||||||||||||||| ||||||||
Sbjct: 560 CTCTCTTACGCGAGCAGATCTTAATCCCATTTCAAGTTTCTCCTGCAACGTTTGTAAATT 501


Query: 241 TTCCAGTGTGATATCTTCAAGATCAGAGCCACCCATTTGCCTG 283
           |||||||||||||||||||||||||||||||||||||||||||
Sbjct: 500 TTCCAGTGTGATATCTTCAAGATCAGAGCCACCCATTTGCCTG 458
```

A third single base gap within a T repeatative motifs

Score = 71.8576 bits (36), Expect = 1.82325e-09
Identities = 43/44 (97%), Gaps = 1/44 (2%), Frame = +1 / -1

```
Query: 282 CATTCGTTGCGCCCCATCCATTTG-TTTTGAAGCAATTCGCACT 324
           |||||||||||||||||||||||| |||||||||||||||||||
Sbjct: 356 CATTCGTTGCGCCCCATCCATTTGTTTTTGAAGCAATTCGCACT 313
```

>lcl|MA_6653 len=41165

Length = 41,165

Score = 297.846 bits (150), Expect = 1.70439e-77
Identities = 188/203 (92%), Gaps = 12/203 (5%), Frame = +1 / -1

```
Query: 324   ACCCCAAATGCAAAGAAGTATCATAGTCACCAAAATATTGATTCTCCTCGTTATTCTCCT 383
             ||||||||||||||||||||||||||||||| ||||||||||||            ||||
Sbjct: 31085 ACCCCAAATGCAAAGAAGTATCATAGTCACCGAAATATTGATTC------------TCCT 31038

Query: 384   CGTTGTTATTCAATTGTAGATTAGCATGGATACTTGAACTTTCCAAAGATTGCACTTCAC 443
             ||||||||||| |||||||||||| |||||||||||||||||||||||||||||||||||
Sbjct: 31037 CGTTGTTATTCGATTGTAGATTAGTATGGATACTTGAACTTTCCAAAGATTGCACTTCAC 30978

Query: 444   AATTTCTTTGTGGCATGGATTCCATCAACAAAGATGCATCTCTTGATGAGCTAACCCCTT 503
             ||||||||||||||||||||||||||||||||||||||||||||||||||||||||||||
Sbjct: 30977 AATTTCTTTGTGGCATGGATTCCATCAACAAAGATGCATCTCTTGATGAGCTAACCCCTT 30918

Query: 504   CTTGTCCAAATATTGTATTCAAC 526
             |||||||||||||||||||||||
Sbjct: 30917 CTTGTCCAAATATTGTATTCAAC 30895
```

Score = 240.358 bits (121), Expect = 3.44558e-60
Identities = 139/145 (95%), Frame = +1 / -1

```
Query: 524   TTTTTATATTATCACTACTTTACATGGTTAATTAACACTATCAAACATGACCACTATAG 583
             ||||||||||||||| ||||||||||||||||||||||||||||||||||||| |||||
Sbjct: 30795 TTTTTATATTATCATTACTTTACATGGTTAATTAACACTATCAAACATGACCGCTATAT 30736

Query: 584   AGGGTAATGTTGCTGCAACTACTTTTGTACATAAGTGATACCGAAAAGATCAAGCATGAT 643
             ||||||||||||||||||||||||||||||||||||||||| ||||||| ||||||||||
Sbjct: 30735 AGGGTAATGTTGCTGCAACTACTTTTGTACATAAGTGATACTGAAAAGACCAAGCATGAT 30676

Query: 644   CATGGATAAGTGGGACTAATTAACC 668
             ||||||||||||||| |||||||||
Sbjct: 30675 CATGGATAAGTGGGATTAATTAACC 30651
```

White spruce genome assembly blast results

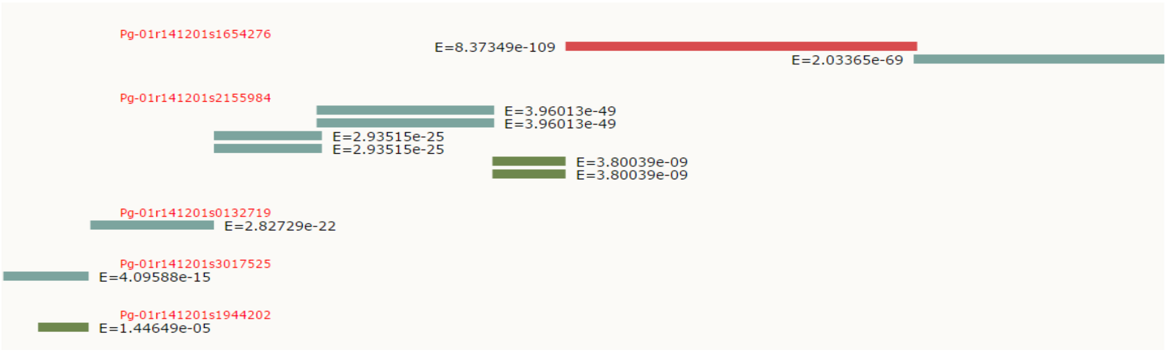

>lcl|Pg-01r141201s3017525 No definition line found

Length = 247,946

Score = 91.6812 bits (46), Expect = 4.09588e-15
Identities = 49/50 (98%), Frame = +1 / -1

```
Query: 1     TTGGAACTGGAATATTCAGAGAGCTTTCCCGAGCAAGAAAATACAACAAG 50
             |||||||||||||||||||||||||||||||||| |||||||||||||||
Sbjct: 13563 TTGGAACTGGAATATTCAGAGAGCTTTCCCGAGCCAGAAAATACAACAAG 13514
```

Two single base gaps within repeatative motifs

>lcl|Pg-01r141201s0132719 No definition line found

Length = 821

Score = 115.469 bits (58), Expect = 2.82729e-22
Identities = 72/74 (97%), Gaps = 2/74 (2%), Frame = +1 / -1

```
Query: 51  CACGAGCTTCCAATTCAAA-GATGCTCCACTTTCATTTGAAAAATTC-TATATCTCGCCA 108
           ||||||||||||||||||| ||||||||||||||||||||||||||| ||||||||||||
Sbjct: 426 CACGAGCTTCCAATTCAAAAGATGCTCCACTTTCATTTGAAAAATTCATATATCTCGCCA 367


Query: 109 CGATCTTCTTCATG 122
           ||||||||||||||
Sbjct: 366 CGATCTTCTTCATG 353
```

>lcl|Pg-01r141201s2155984 No definition line found

Length = 7,038

Score = 125.381 bits (63), Expect = 2.93515e-25
Identities = 63/63 (100%), Frame = +1 / -1

```
Query: 122  CCTGCACATATGGTCGAGTTCTTCACATTGCTTTTTCAGCTCTGCGAATTCGCTCTGATC 181
            ||||||||||||||||||||||||||||||||||||||||||||||||||||||||||||
Sbjct: 4016 CCTGCACATATGGTCGAGTTCTTCACATTGCTTTTTCAGCTCTGCGAATTCGCTCTGATC 395


Query: 182  CGC 184
            |||
Sbjct: 3956 CGC 3954
```

Score = 204.675 bits (103), Expect = 3.96013e-49
Identities = 103/103 (100%), Frame = +1 / -1

```
Query: 181  CTCTCTTACGCGAGCAGATCTTAATCCCATTTCAAGTTTCTCCTGCAACGTCTGTAAATT 240
            ||||||||||||||||||||||||||||||||||||||||||||||||||||||||||||
Sbjct: 3841 CTCTCTTACGCGAGCAGATCTTAATCCCATTTCAAGTTTCTCCTGCAACGTCTGTAAATT 3782


Query: 241  TTCCAGTGTGATATCTTCAAGATCAGAGCCACCCATTTGCCTG 283
            |||||||||||||||||||||||||||||||||||||||||||
Sbjct: 3781 TTCCAGTGTGATATCTTCAAGATCAGAGCCACCCATTTGCCTG 3739
```

A third single base gap within a T repeatative motifs

Score = 71.8576 bits (36), Expect = 3.80039e-09
Identities = 43/44 (97%), Gaps = 1/44 (2%), Frame = +1 / -1

```
Query: 282  CATTCGTTGCGCCCCATCCATTTG-TTTTGAAGCAATTCGCACT 324
            |||||||||||||||||||||||| |||||||||||||||||||
Sbjct: 3637 CATTCGTTGCGCCCCATCCATTTGTTTTTGAAGCAATTCGCACT 3594
```

>lcl|Pg-01r141201s1654276 No definition line found

Length = 964

Score = 402.911 bits (203), Expect = 8.37349e-109
Identities = 203/203 (100%), Frame = +1 / +1

```
Query: 324 GTTGAATACAATATTTGGACAAGAAGGGGTTAGCTCATCAAGAGATGCATCTTTGTTGAT 383
           ||||||||||||||||||||||||||||||||||||||||||||||||||||||||||||
Sbjct: 265 GTTGAATACAATATTTGGACAAGAAGGGGTTAGCTCATCAAGAGATGCATCTTTGTTGAT 324


Query: 384 GGAATCCATGCCACAAAGAAATTGTGAAGTGCAATCTTTGGAAAGTTCAAGTATCCATGC 443
           ||||||||||||||||||||||||||||||||||||||||||||||||||||||||||||
Sbjct: 325 GGAATCCATGCCACAAAGAAATTGTGAAGTGCAATCTTTGGAAAGTTCAAGTATCCATGC 384


Query: 444 TAATCTACAATTGAATAACAACGAGGAGAATAACGAGGAGAATCAATATTTTGGTGACTA 503
           ||||||||||||||||||||||||||||||||||||||||||||||||||||||||||||
Sbjct: 385 TAATCTACAATTGAATAACAACGAGGAGAATAACGAGGAGAATCAATATTTTGGTGACTA 444


Query: 504 TGATACTTCTTTGCATTTGGGGT 526
           |||||||||||||||||||||||
Sbjct: 445 TGATACTTCTTTGCATTTGGGGT 467
```

Score = 272.075 bits (137), Expect = 2.03365e-69
Identities = 143/145 (98%), Frame = +1 / +1

```
Query: 524 GGTTAATTAGTCCCACTTATCCATGATCATGCTTGATCTTTTCGGTATCACTTATGTACA 583
           ||||||||| ||||||||||||||||||||||||| ||||||||||||||||||||||||
Sbjct: 568 GGTTAATTAATCCCACTTATCCATGATCATGCTTGGTCTTTTCGGTATCACTTATGTACA 627


Query: 584 AAAGTAGTTGCAGCAACATTACCCTCTATAGTGGTCATGTTTGATAGTGTTAATTAACCA 643
           ||||||||||||||||||||||||||||||||||||||||||||||||||||||||||||
Sbjct: 628 AAAGTAGTTGCAGCAACATTACCCTCTATAGTGGTCATGTTTGATAGTGTTAATTAACCA 687


Query: 644 TGTAAAGTAGTGATAATATAAAAAA 668
           |||||||||||||||||||||||||
Sbjct: 688 TGTAAAGTAGTGATAATATAAAAAA 712
```
